# Percent Reduction in Transverse Rupture Strength of Metal Matrix Diamond Segments Analysed via Discrete-Element Simulations

**DOI:** 10.3390/ma11061048

**Published:** 2018-06-20

**Authors:** Xiuyu Chen, Guoqin Huang, Yuanqiang Tan, Yiqing Yu, Hua Guo, Xipeng Xu

**Affiliations:** 1 Institute of Manufacturing Engineering, Huaqiao University, Xiamen 361021, China; yu@hqu.edu.cn (X.C.); tanyq@hqu.edu.cn (Y.T.); yyqing@hqu.edu.cn (Y.Y.); guoh1214@hqu.edu.cn (H.G.); xpxu@hqu.edu.cn (X.X.); 2 MOE Engineering Research Center for Machining of Brittle Materials, Huaqiao University, Xiamen 361021, China; 3 Fujian Engineering Research Center of Intelligent Manufacturing for Brittle Materials, Fujian 361021, China

**Keywords:** diamond segment, transverse rupture strength, discrete-element model, metal matrix, bonding strength

## Abstract

The percent TRS reduction, D_TRS_, which is the percent reduction of the transverse rupture strength of metal matrix diamond segments with or without diamonds, is a key metric for evaluating the bonding condition of diamonds in a matrix. In this work, we build, calibrate, and verify a discrete-element simulation of a metal matrix diamond segment to obtain D_TRS_ for diamond segments with various diamond-grain sizes, concentrations, and distributions. The results indicate that D_TRS_ increases with increasing diamond-grain concentration and decreases with increasing diamond-grain size. Both factors can be explained by the total diamond contact length, the increase of which causes the increase in D_TRS_. The distribution of diamond grains in segments also strongly influences the increase of D_TRS_. The use of D_TRS_ as a metric to assess the bonding condition of diamonds in matrixes is not valid unless the diamond-grain size, concentration, and distribution and total diamond contact length are the same for all diamond segments under consideration.

## 1. Introduction

Metal matrix diamond segments are key components for metal matrix diamond tools, which are widely used in industrial applications. A diamond segment is composed of a metal matrix containing diamonds. The main role of the matrix is to bond the diamonds to their segment for as long as possible during use, which is commonly referred to as “diamond retention”. The transverse rupture strength (TRS) is an important factor for assessing diamond retention and the wear performance of diamond segments [1,2]. In particular, the percent TRS reduction, D_TRS_, which represents the percent reduction in TRS between a segment without diamonds and one with diamonds, is used to determine the bonding condition between diamond and metal matrix [2,3,4].

A large D_TRS_ indicates that diamonds are weakly bonded to the matrix. Previous studies experimented with various ingredients for the metal matrix and various diamond coatings [2,3,4,5] and found that D_TRS_ sometimes changes unexpectedly, although the metal matrix, diamond-grain size, and diamond concentration were the same in each experiment. In other words, D_TRS_ becomes unexpectedly invalid during applications. The reason behind this unexpected change is tentatively attributed to the assumption used in deriving D_TRS_, that all diamonds in segments are exactly the same and uniformly distributed. Although it is true that the diamond distribution in segments is difficult to control with precision, it is not certain whether the diamond-grain distribution does affect D_TRS_.

In addition to the diamond-grain distribution, the diamond-grain concentration and size have also not been fully studied, and all these factors are difficult to control in experiments. To resolve this difficulty, we use the discrete-element method (DEM) to simulate these systems. Cundall [6,7] first applied the DEM to analyse the behaviour of granular materials and, since then, it has been widely applied to many other materials, such as concrete [8], ceramics [9], and SiC [10]. Thus, in this work, we first build, calibrate, and verify a DEM simulation of a Co-based metal matrix diamond segment, and then analyse the percent TRS reduction of DEM-simulated diamond segments with different diamond-grain concentrations, sizes, and distributions.

## 2. Materials and Experimental Details

### 2.1. Fabrication of Diamond Segments

A Co-based matrix, the composition of which was 70Co–27Cu–3Sn (wt %), was used as the metal matrix in this work. Diamond grains were purchased from HuangHe Diamond Limited Company, Henan, China. Diamond grains and metal powders of Co, Cu, and Sn were mixed and blended for 120 min via a rotary mixer (SYH01, Chunlai Machinery, Changzhou, China), then poured into a graphite mould, and finally sintered at a temperature of 810 °C with a hold time of 2 min and a hold pressure of 15 MPa on an automatic hot-pressing sinter machine (SMVBC, Golden Highway, Zhengzhou, China), which was installed with an infrared sensor (RS-WD-HW-120, Jiandakeren, Shandong, China) for temperature monitoring.

### 2.2. The Three-Point Bending Tests and Compression Tests

Diamond segments with dimensions of 30 mm × 12 mm × 6 mm were fabricated for three-point bending tests and dimensions of 6 mm × 6 mm × 6 mm were fabricated for compression tests. The TRS and Young’s modulus (Ec) were determined by a three-point bending test in which the bending span was set to 25 mm. The uniaxial compressive strength (UCS) was determined by compression tests. Both tests were performed on an Instron5569 tester (Instron, Boston, MA, USA). For each condition of test, 10 specimens were repeated to obtain the averaged value. A 3D digital microscope (KH8700, HIROX, Tokyo, Japan) was used to observe the fracture failure of the specimen after being tested.

### 2.3. Single Grit Shearing Test

**Figure 1:**
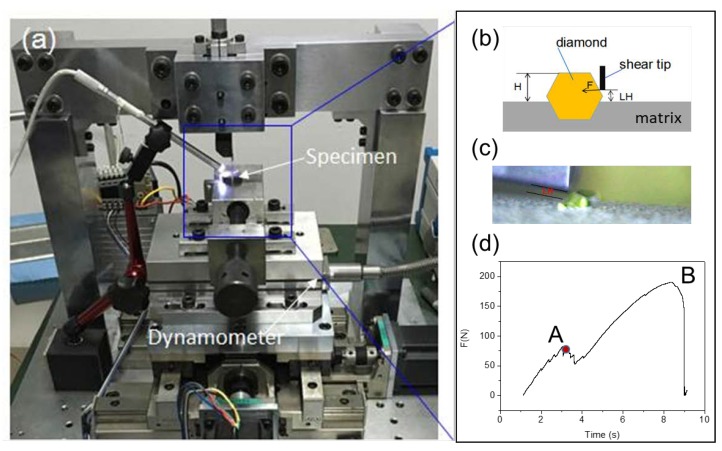
Illustration of shearing test: (**a**) shearing test device; (**b**) schematic of shearing; (**c**) real shearing; (**d**) shearing force signal.

A single grit shear test was carried out on a self-made shear device illustrated in Figure 1. The force was measured by a dynamometer (9265B, Kistler, Jonsered, Switzerland), as shown in Figure 1a. In Figure 1b, the value of LH, which is the distance between the shear point and the bonded level of diamond, was set to 100 μm and the shear speed VS was set to 0.5 mm/s. A shearing force signal shown in Figure 1d shows that the shearing force F increased linearly at the beginning, dropped slightly when the interfacial debonding occurred, as point A shown in Figure 1d, and then continued to increase to its maximum value when the diamond was rolled out. The critical force, which is the force at the time the interfacial debonding occurred, was used to calibrate the microscopic parameters of the diamond/matrix boundary.

For the calibration of diamond/matrix boundary, the critical force of 10 sintered diamonds with an exposed height H of 230 μm was tested and averaged. To certify the diamond/matrix boundary, the critical force of 10 sintered diamonds with an exposed height H of 280 μm was tested and averaged.

## 3. Establishment, Calibration, and Verification of Discrete-Element Model

The DEM simulation was implemented by using the software of PFC^2D^ (Particle Flow Codes in two dimensions). The DEM model was composed of particles and the bonds between them. In such a model, a contact bond is an interaction between two particles because the particles themselves are deformable, and a parallel bond is an approximation of the physical behaviour of a bond substance between particles [6,11].

**Figure 2:**
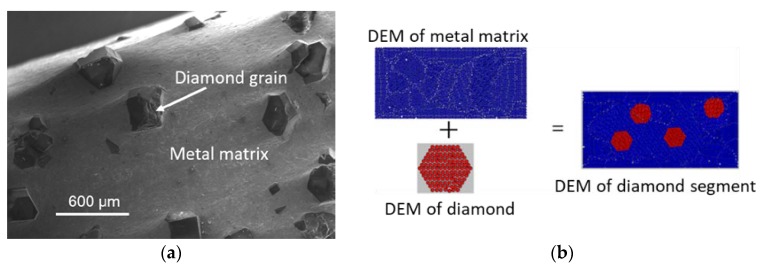
Illustration of establishing the DEM of a diamond segment: (**a**) the morgraphy of diamond segment (observed by Scanning Electron Microscope); (**b**) DEM of a diamond segment.

A diamond segment consists of a metal matrix and diamond grains, as shown in Figure 2a. Thus, the DEM model of a diamond segment consists of the model of a metal matrix and a model of diamond. The DEM model of the metal matrix is directly represented by spherical particles. The DEM model of the diamond grain is indirectly represented by a hexagonal cluster, which is composed of spherical particles. In other words, the DEM models of the metal matrix and the diamonds should be built individually at first. Then, the model of the diamond grains should be added to the matrix model to form the DEM model of the diamond segment (see Figure 2b).

In DEM, the microcosmic parameters of the particles in the DEM model directly determine the macrophysical and macromechanical parameters of the DEM simulation. The manner in which these microcosmic parameters are obtained is the premise of the DEM simulation. Reference [12] indicates that they can be calibrated based on a molecular dynamics (MD) simulation, but this is difficult to do for diamond segments because of the scale problem. In fact, a more general calibration methodology is calibrating these parameters by the inversion method, in which the macrocosmic outcome of large-scale DEM simulations is compared with bulk attempts [11,13,14,15].

Therefore, based on the above analyses, this section details the DEM models for a metal matrix in Section 3.1, for diamonds in Section 3.2, and for diamond segment in Section 3.3. The model of the diamond segment is verified in Section 3.4.

### 3.1. Discrete-Element Model of Metal Matrix

**Figure 3:**
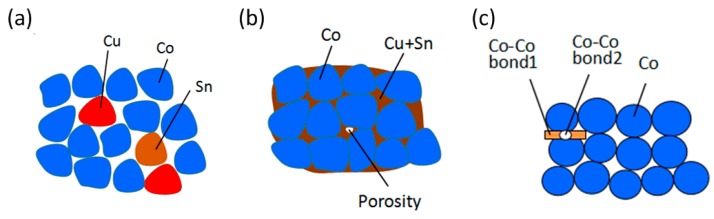
Illustration of Co–Cu–Sn-based metal matrix: (**a**) before sintering, (**b**) after sintering, and (**c**) DEM Co–Cu–Sn metal matrix.

As the Co-based matrix used as the metal matrix in this work was 70Co–27Cu–3Sn in wt %, the matrix was composed of Co, Cu, and Sn powder particles. Typically, diamond segments are fabricated by hot sintering, which is a solid-liquid process. Because the sintering of the metal matrix diamond segment is also done by pressing sintering, the metal particles are squeezed together. In the DEM of a metal matrix, the particles only represent the skeleton substance, such as Co, whereas the bonding substance, such as Cu or Sn, is represented by parallel bonds between particles. In this work, the contact bond was also used in the matrix model to describe the elasticity of the metal matrix. Thus, two types of bonds existed in the DEM of the Co-based matrix used in this work, as shown in Figure 3: the Co–Co bond 1 is a parallel bond, and the Co–Co bond 2 is a contact bond.

**Figure 4:**
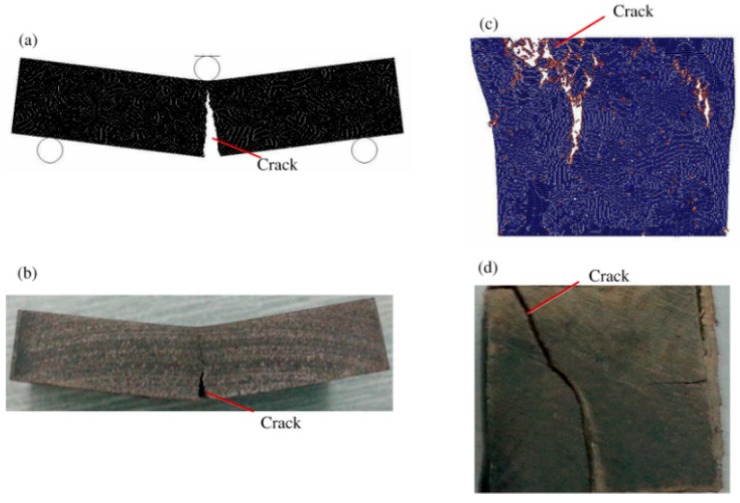
Failure of Co-based metal matrix after being tested: (**a**,**c**) by DEM simulation; (**b**,**d**) by experiment.

To invert the microscopic parameters of the metal matrix, the three-point bending test and compression test were calculated by using the DEM model. The simulated TRS, UCS, and Ec were compared with the corresponding experimental results, the experimental details of which are presented in Section 2. When the simulated results match the experimental results with an error of less 10%, the microcosmic parameters of the Co-based metal matrix DEM model were calibrated. The matching results are listed in Table 1. Figure 4 also shows the comparison of segment failure by ways of experiment and simulation. It can be seen that the features of fracture failure by experiment were identical to that by simulation.

### 3.2. Discrete-Element Model of Diamond

**Figure 5:**
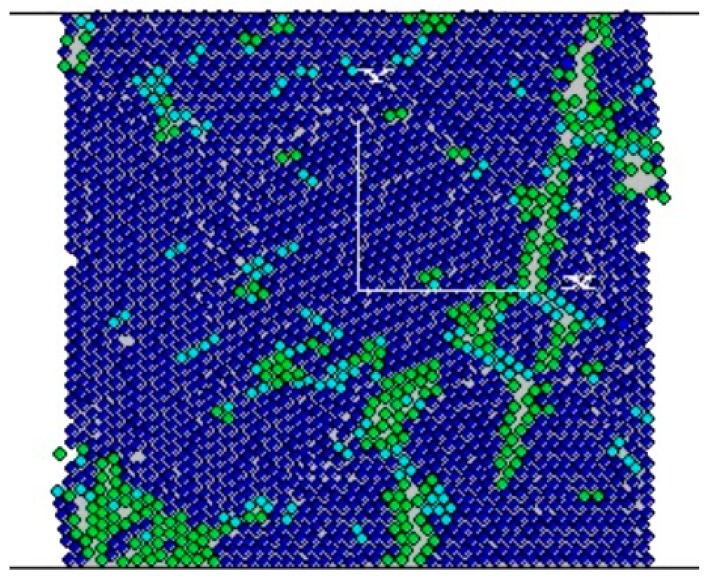
Compression test simulation of the diamond model.

Due the hexagonal shape of diamonds, the DEM of a diamond grain is built by a cluster of particles bonded in hexagonal shape. The particle size here was 0.023 mm. As a diamond is a typical brittle material, only the parallel bond was used in the model to constrain the particles [8,9,10]. The microcosmic parameters of the diamond model were inverted by a uniaxial compression test, as shown in Figure 5. The values of Young’s Ec, Poisson’s ratio γ, and the UCS obtained by simulation were compared with the corresponding values from [15], as summarized in Table 2.

### 3.3. Discrete-Element Model of the Diamond Segment

The DEM of a diamond segment was built by adding the diamonds’ DEM to the DEM of the metal matrix and then adding bonds to constrain the diamonds and the matrix into a block. The bond between the diamond and the matrix is called as diamond/matrix boundary. As the diamond segment is a brittle composite material with a certain elasticity, both the parallel and contact bonds were combined to describe the bonding condition of the diamond/matrix boundary.

Besides the microscopic parameters of DEMs of the matrix and diamond, the microscopic parameters also need to be calibrated. In this work, the microscopic parameters of the diamond/matrix boundary bond were calibrated by single diamond shear testing. The experiment condition is detailed in Section 2. The calibration was done in three steps as follows:

Step 1: A DEM model of the Co-based metal matrix diamond segment was made. The values of the parameters for the metal matrix and diamond were set according to the results of calibrations in Section 3.1 and Section 3.2. The initial values of the parameters of the diamond/matrix boundary were then assigned.

**Figure 6:**
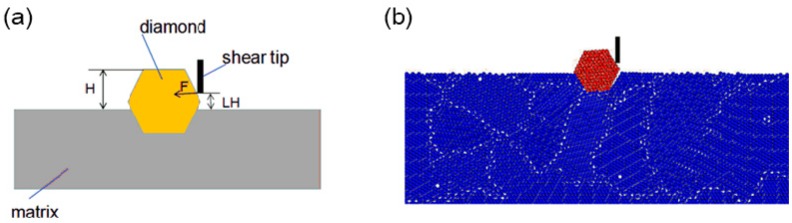
Single diamond shearing test: (**a**) schematic diagram; (**b**) simulation by DEM.

Step 2: The shearing test in the DEM was simulated, as shown in Figure 6, in which the exposure height H was set as 230 μm. Comparing the simulated critical force with the experimental critical force, when the simulation result matched the experimental result with an error of less 10%, as shown in Table 3, the microcosmic parameters of the diamond/matrix boundary were inverted.

Step 3: After inverting the microscopic parameters, the exposure height H of 280 μm was used to verify the accuracy of the diamond/matrix boundary’s microcosmic parameters. The shear force by simulation and experiment are also compared in Table 3.

### 3.4. Verification of Discrete-Element Model of the Diamond Segment

Based on the above work, the DEM of the diamond segment was built and all of its microscopic parameters were calibrated, which are summarized in Table 4.

To verify the validity of the diamond segment’s DEM, a three-point bending test was carried out. Here, diamond grains with a size of 550 μm (30/40 US mesh) and a concentration of 50% were used. It should be noted that in the diamond industry, the 25% volume of diamonds by cm^3^ or 0.88g (4.4 Carat) of diamonds by cm^3^ is defined as the 100% concentration of diamonds in the segment [17]. The TRS obtained by simulation and experiment are compared in Table 5, the error of which is within the satisfactory value of less 10%.

## 4. Simulation of Percent Reduction of TRS

The percent TRS reduction, D_TRS_, is generally used to describe the retention ability of diamonds in diamond segments. Thus, the D_TRS_ was calculated by: D_TRS_ = (σ_0_ − σ)/σ_0_ × 100%, where σ_0_ is the TRS of the metal matrix and σ is the TRS of the diamond segment with diamonds.

For a diamond segment with different ingredients under the same sintering conditions, when the concentration and the sizes of the diamonds in the segment were kept the same, a smaller D_TRS_ equated to a generally stronger diamond retention [18]. In order to study the feasibility of this approach, the DEM model with the microcosmic parameters obtained from Section 4 was applied to study the factors that influence the D_TRS_. To do this, D_TRS_ was calculated by the DEM simulation in which diamond concentration, diamond size, and diamond distribution were varied. In this section, the dimension of the segment’s DEM model was 30 mm × 6 mm.

### 4.1. Effect of Diamond Concentration

**Figure 7:**
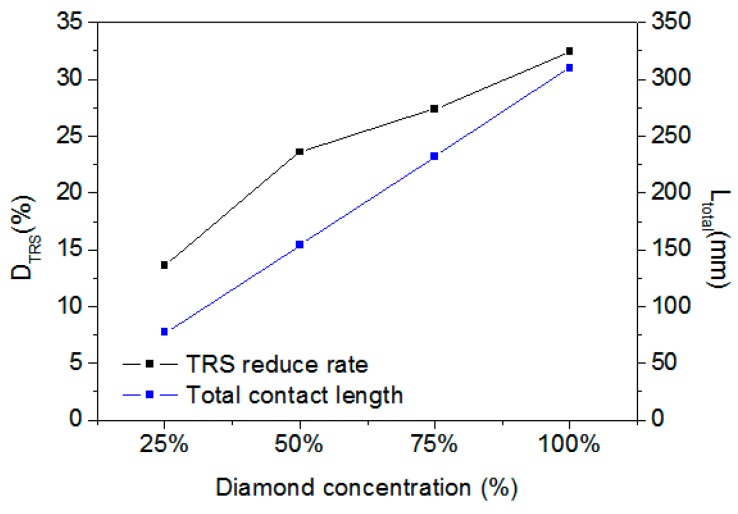
D_TRS_ and total diamond contact length L_total_ versus diamond concentration.

To determine how diamond concentration affects D_TRS_, diamonds were added to the Co-based matrix with concentrations of 25%, 50%, 75%, and 100%. The simulated D_TRS_ are plotted in Figure 7. It can be found that the D_TRS_ increases with increasing diamond concentration.

As the dimension of the segment’s DEM was 30 mm × 6 mm for the four concentrations above, their corresponding diamond weights were 0.36, 0.72, 108, and 1.44 carat/cm^3^, and the number of diamond grains were 43, 88, 129, and 172. As a typical of composite material, diamonds can be considered as a heterogeneous material to the matrix. Due to the DEM simulation in this work being in 2D mode, the total diamond contact length was used to quantitate the contacting boundary of the diamonds in the matrix. As the diamond-grain size was 550 µm, the total diamond contact length L_total_ for the four concentrations were 77, 154, 231, and 308 mm, respectively. The relationship between D_TRS_ and L_total_ is presented in Figure 7. It can be easily concluded that D_TRS_ increases nearly linearly with L_total_.

### 4.2. Effect of Diamond-Grain Size

**Figure 8:**
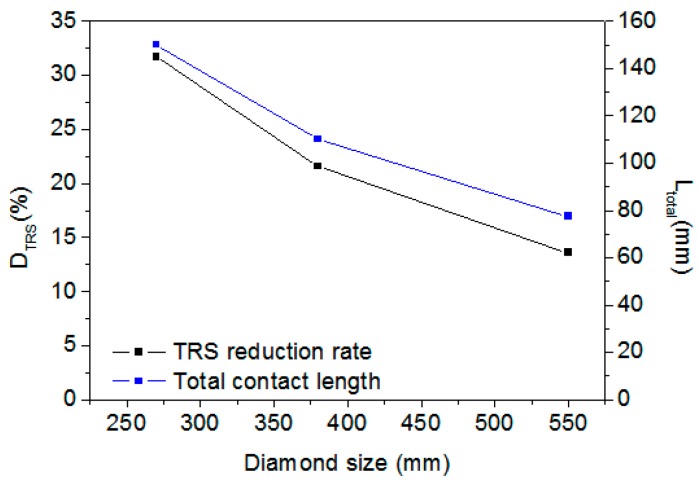
Percent TRS reduction D_TRS_ and total diamond boundary length L_total_ versus diamond size.

To determine the effect of diamond-grain size, diamond segments with the same grain concentration of 25% but with different diamond-grain sizes of 550 µm (30 US mesh), 380 µm (40 US mesh), and 270 µm (50 US mesh) were simulated. Figure 8 presents the D_TRS_ and L_total_ versus the increase of diamond-grain size. Figure 8 also shows that, with the same grain concentration, D_TRS_ decreases with increasing diamond size.

As the dimension of the segment was 30 mm × 6 mm, the number of diamond grains was 44, 92, and 180, and L_total_ were 77, 110, and 150 mm, respectively, for the three types of diamond segments. It can be found that with the same diamond-grain concentration, the number of diamond grains and L_total_ in the DEM model depend on the diamond-grain size. From Figure 8, it also can be found that D_TRS_ increases linearly with total contact length, which agrees with the result shown in Figure 7.

To further check the effect of grain size, four diamond segments were designed with the same total contact length. This was done by using the same total number of diamond grains and the same diamond distribution, but with different diamond-grain sizes, as listed in Table 6. The total contact length was set as 110 mm, and the total number of diamonds was set as 92. Then, the diamond-grain concentrations were calculated to be 25%, 29.7%, 29.7%, and 32% for segments S1–S4, respectively (see Table 6).

**Figure 9:**
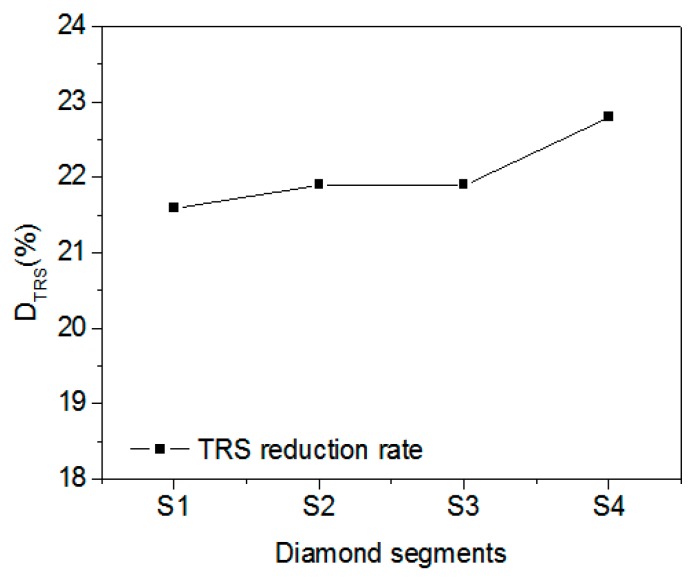
Percent TRS reduction for the various diamond segments (see Table 6).

Next, D_TRS_ was calculated for the four segments (Figure 9 and Table 6). Ordering the segments from smallest D_TRS_ to largest gives S1 < S2 = S3 < S4. Based on Table 6, ordering the segments from smallest diamond-grain concentration to largest gives the same result: S1 < S2 = S3 < S4. By comparing these two ordered lists, it can be concluded that: (i) D_TRS_ generally increases with increasing grain concentration, which is consistent with the result discussed in the Section 3.1; and (ii) for the same L_total_ and diamond-grain concentration, D_TRS_ is independent of diamond-grain size. The diamond-grain concentration and total contact length of diamonds strongly impact the D_TRS_. The total contact length of the diamonds corresponds with the contact surface area between the diamond grains and the matrix in actual three-dimensional diamond segments. It is reasonable that with the increase of diamonds in the matrix, the TRS reduction increases.

### 4.3. Effect of Diamond Distribution

**Figure 10:**
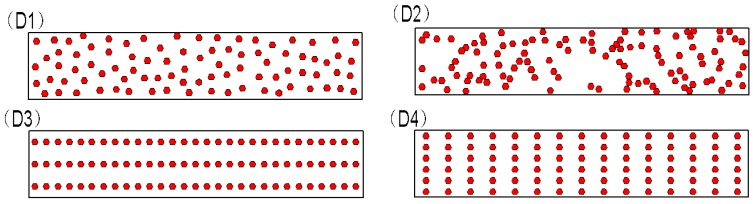
Diamond distributions D1–D4. See text for details.

**Figure A1:**
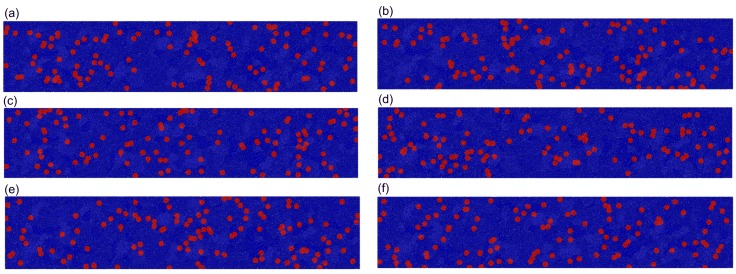
Six different random distributions of diamond in segment.

**Figure A2:**
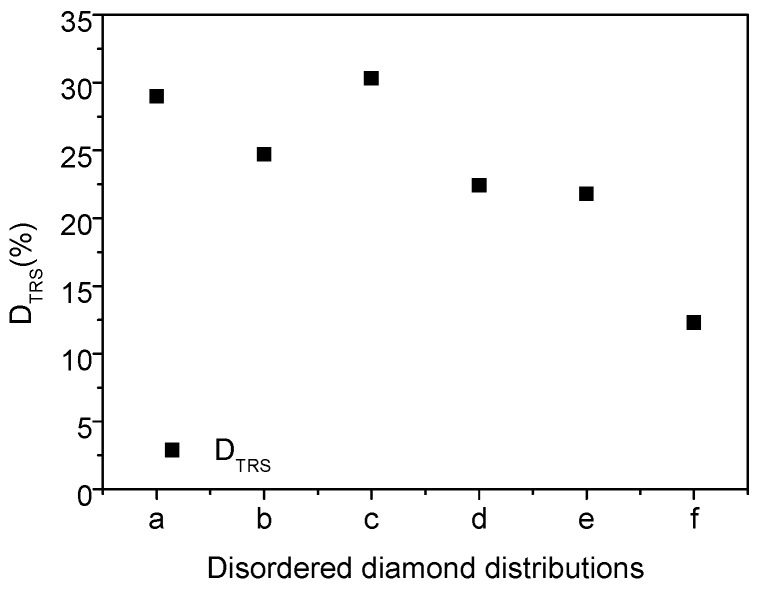
Disorderly distribution VS reduce rate of TRS.

To investigate the effect of diamond-grain distribution with the same diamond-grain concentration and diamond-grain size, segments with four diamond-grain distributions were designed, which are shown in Figure 10. To avoid the effect of diamond-grain size, diamond-grain concentration, and total contact length on D_TRS_, in the Figure 10, the diamond particle size was 550 μm and the concentration was 50% (with 90 diamonds in each segment) for all four samples. The four distributions are based on the following considerations:

(1) As described in the introduction, a uniform distribution presupposes using the percent TRS reduction to assess diamond retention. Thus, distribution D1 is a uniform distribution.

(2) In real sintered diamond tools, diamonds are distributed by mixing diamond grains with metal matrix powders in a rotary machine before sintering. In theory, diamonds can be uniformly distributed given sufficient mixing time. However, diamonds are distributed with a random distribution and some even reunite, as shown in Figure 2a. We set six random distributions for diamonds in a segment, as shown in Figure A1 in Appendix. From Figure A2 in Appendix, it shows that the different distributions correspond to different D_TRS._ Thus, distribution D2 is a random distribution, which is the same as the distribution (a) in Figure A1 in Appendix.

(3) For the sake of comparison, two ordered distributions D3 and D4 were designed. D3 has three rows, and D4 has six rows (the number and the size of the diamonds in D3 and D4 are the same).

The TRS of the matrix, which is without diamonds, is 1046 MPa, as shown in Table 1, and the results for D_TRS_ of the segments with the different diamond-grain distributions are listed and compared in Table 7. The 5.1% difference in D_TRS_ of distributions D1 and D2 is caused by the spatial difference in distributions D1 and D2. For the ordered distributions, the difference of 18% between D_TRS_ for D3 and D4 is caused by changing the number of rows from three to six. It can be concluded that the diamond-grain distribution is also a key factor determining the D_TRS_.

**Figure 11:**
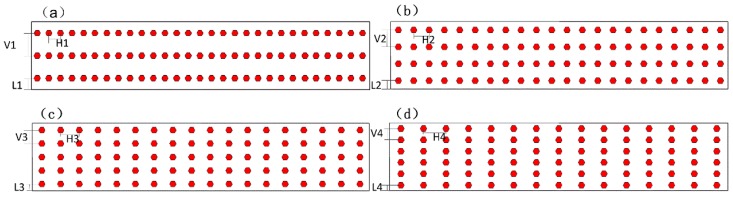
Different order distributions of diamonds in the segment: three rows (**a**), four rows (**b**), five rows (**c**), and six rows (**d**).

The comparison between D3 and D4 indicates that different orderly arrangements also have a significant effect on the TRS reduction rate. When the number of rows changed from three to six, the TRS reduction rate changed from 12.3% to 30.3%. In order to study the effect further, two more kinds of ordered distributions were taken into account, as shown in Figure 11 (which is the same as Figure 10, in which the dimension segment was 30 mm × 6 mm, the diamond size was 550 μm, and the number of diamonds in each segment was 90). The distances of two diamonds were different: V1–V4 in Figure 11 were 2 mm, 1.5 mm, 1.2 mm, and 1 mm, respectively; H1–H4 were 1.04 mm, 1.36 mm, 1.67 mm, and 2 mm, respectively; and L1–L4 were 1 mm, 0.75 mm, 0.6 mm, and 0.5 mm, respectively.

**Figure 12:**
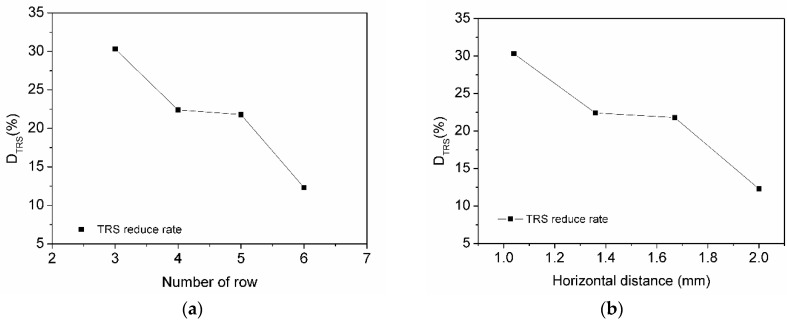
Order distribution versus reduction rate of TRS. (**a**) Effect of row number; (**b**) effect of horizontal distance.

The effects of the row’s number and horizontal distance on D_TRS_ are presented in Figure 12. It can be seen that the D_TRS_ decreased with the increase of either row’s number or the horizontal distance.

L1–L4 is the distance from specimen edge to the nearest diamond in distributions (**a**–**d**) in Figure 11. L1 is the greatest and L4 is the least in L1–L4. The bond of the matrix/diamond boundary is weaker than the bond of the matrix/matrix. According to the features of the composite material, the closer the weak bond is to the edge, the easier it is for the material to be destroyed. However, from Figure 12, we can see the D_TRS_ of the segment with three rows is larger than the segment with six rows, which means that the D_TRS_ of the segment with L1 (1 mm) is larger than the segment with L4 (0.5 mm), and the segment with L1 is easier to be destroyed. Thus, we can find that the distance from the specimen edge to the nearest diamond interface has less affect than the distance of the changed horizontal direction.

### 4.4. Discussion

These simulation results led us to conclude that the diamond-grain size, concentration, and distribution in segments strongly affect the percent of TRS reduction.

However, note that all of the above simulations were implemented by DEM with the same matrix and the same diamond-matrix bonding conditions because all the microscopic parameters in the DEM models for the metal matrix, diamond clusters, and diamond segments were fixed after being calibrated. In other words, the differences obtained for D_TRS_ in Section 4.1, Section 4.2 and Section 4.3 were induced only by the different diamond distributions, rather than by the diamond bonding conditions with the matrix. This observation can reasonably explain the unexpected change in D_TRS_ while evaluating the diamond bonding condition by TRS. Therefore, D_TRS_ is not a reasonable metric to evaluate the bonding condition of diamonds in segments unless the grain size, concentration, and distribution of diamonds in the segments are the exactly same.

## 5. Conclusions

The major factors affecting the percent of TRS reduction, D_TRS_, of diamond segments are the diamond-grain size, concentration, and distribution within the diamond segment. These factors were analysed by DEM simulation, which also considered the diamond total contact length L_total_ to explain the results.

The results show that D_TRS_ for diamond segments increases with diamond-grain concentration but decreases with diamond-grain size. The percent of TRS reduction increases with the increase of the L_total_ in the segment. For a given L_total_, diamond-grain concentration, and diamond-grain distribution, the diamond grains of differing sizes have no effect on D_TRS_. However, the spatial distribution of the diamond grains with the segment strongly affects the D_TRS_. Finally, the D_TRS_ is not a reasonable metric for evaluating the bonding conditions of diamonds in segments unless the diamond-grain size, concentration, and distribution are the same in all segments under consideration.

## Figures and Tables

**Table 1:** Values of UCS, Ec, and TRS of metal matrix obtained by experiment and DEM simulation.

Mechanical Properties	Co-Based Metal Matrix (Segment without Diamonds)		
	Experimental Results	Simulation Results	Error
UCS/MPa	1681	1842	9.6%
Ec/GPa	13.8	12.8	7.2%
TRS/MPa	1120	1046	4.2%

**Table 2:** Main mechanical properties of the diamond obtained by the DEM simulation and ref. [15].

Mechanical Properties	Values from [16]	Values Simulated by DEM Model
Young’s modulus Ec (GPa)	900–1000	940
Poisson’s ratio γ	0.069–0.12	0.095
UCS (MPa)	4500–5800	4853

**Table 3:** Critical force of diamond obtained by experiment and DEM simulations.

Exposure Height (µm)	Critical Force of Shearing Test, F (N)		Error (%)
	By Experiment (N)	By Simulation (N)	
230	70.5	71.1	0.9
280	67.3	68	1.0

**Table 4:** The values of microcosmic parameters of the diamond segment.

	Values of Microcosmic Parameters		
	Matrix	Diamond	Diamond/Matrix Boundary
Particle density (kg/m^3^)	8900	3500	/
Particle contactmodulus, Ec (GPa)	1.3 × e^10^	3 × e^11^	/
Particle stiffnessratio, kn/ks	1	1	/
Particle friction coefficient	0.8	0.1	/
Isotropics Stress, (Pa)	−2.0 × e^7^	−5.0 × e^7^	/
Radius multiplier of parallel bond	1	1	1
Elasticity modulus of parallel bond (Pa)	1.3 × e^9^	11 × e^11^	4 × e^7^
Normal strength of parallel bond (Pa)	3 × e^8^	1 × e^10^	4 × e^3^
Shear strength of parallel bond (Pa)	3 × e^8^	1 × e^10^	4 × e^3^
Normal strength of contact bond (Pa)	6 × e^7^	/	5 × e^3^
Shear strength of contact bond (Pa)	6 × e^7^	/	5 × e^3^

**Table 5:** TRS of the diamond segment: results of experiment and DEM simulations.

Diamond Size (US Mesh)	Concentration (%)	TRS			Ec		
		Simulation (MPa)	Experiment (MPa)	Error (%)	Simulation (N)	Experiment (N)	Error (%)
30/40	50	743	786	5.5	17.4	15.9	9.4

**Table 6:** Properties of diamond segments with the given number of diamonds of the given grain size.

Diamond Segments	Number of Diamonds with Given Grain Size			Total Contact Length (mm)	Diamond Concentration (%)	D_TRS_ (%)
	550 µm	380 µm	270 µm			
S1	0	92	0	110	25	21.6
S2	29	31	32	110	29.7	22.0
S3	23	32	37	110	29.7	22.0
S4	43	0	49	110	32	22.8

**Table 7:** Percent TRS reduction for different diamond distributions.

Diamond Distribution	TRS (MPa)	Percent TRS Reduction (%)
D1	788	24.7
D2	734	29.8
D3	729	30.3
D4	916	12.3
